# Investigation of the interplay of PCSK9, cardiac dynamics, oxidative stress in coronary artery disease: case-control study

**DOI:** 10.3389/fendo.2025.1494438

**Published:** 2025-04-28

**Authors:** Anmar Hussein Lafta, Hamidreza Shiri, Mahsa Iraji, Amin Karimpour, Mahboobe Sattari, Monireh Rahimkhani, Nahid Einollahi, Ghodratollah Panahi

**Affiliations:** ^1^ Department of Clinical Laboratory Sciences, Faculty of Allied Medical Sciences, Tehran University of Medical Sciences, Tehran, Iran; ^2^ Department of Clinical Biochemistry, Faculty of Medicine, Tehran University of Medical Sciences, Tehran, Iran; ^3^ Faculty of Allied Medical Sciences, Tehran University of Medical Sciences, Tehran, Iran

**Keywords:** PCSK9, oxidative stress, myocardial infarction, ox-LDL, EF%

## Abstract

**Background:**

PCSK9 plays a key role in raising LDL-C levels, which contributes to heart attacks (MI). However, studies show that about half of MI patients have normal LDL-C levels. This study aims to explore the link between PCSK9, heart function, and oxidative stress markers in MI patients.

**Methods:**

This investigation was carried out at Tehran Heart Centre Hospital on healthy individuals (n=63) and patients (n=63) with MI who had a coronary artery block above 50% (CAB > 50%). Oxidative stress (OS) parameters, such as total antioxidant capacity (TAC), malondialdehyde (MDA), myeloperoxidase (MPO), superoxide dismutase (SOD), catalase (CAT), and glutathione peroxidase (GPx) activity, PCSK9, oxidized Low-density lipoprotein (ox-LDL), high-sensitivity cardiac troponin I (hs-cTnI), and hs-CRP are assessed. Indeed, biochemical parameters and EF% were measured.

**Results:**

Higher EF% (>37.5%), TAC (>1.05 mmol Fe²^+^;/L), GPx (>16.48 mU/mL), CAT (>11.32 nmol/min/mL), and SOD (>297.16 U/mL) were linked to a lower risk of CAB > 50%. In contrast, higher MDA (>32.07 nmol/mL), MPO (>17.77 U/L), hs-CRP (>5.5 mg/L), and ox-LDL (>64.87 μg/L) were associated with a higher risk. There was no significant difference in PCSK9 and LDL-C levels between groups. EF% was positively linked to SOD but negatively related to MDA, MPO, ox-LDL, hs-cTnI, and hs-CRP. Ox-LDL correlated positively with MPO but negatively with TAC, CAT, and GPx. PCSK9 showed a positive relationship with MDA. The best markers for CAB > 50% diagnosis were ox-LDL (AUC = 83.22, cut-off > 63.35 μg/L), EF% (AUC = 82.35, cut-off < 46.25%), and hs-cTnI (AUC = 81.3, cut-off > 0.265 ng/mL).

**Conclusion:**

While PCSK9’s role in MI through LDL-C is well known, its impact on inflammation and oxidative stress may also be important, even when LDL-C and PCSK9 levels are normal. Additionally, ox-LDL and EF% are better indicators of CAB > 50% than hs-cTnI.

## Introduction

Coronary artery disease (CAD) is one of the most prevalent cardiovascular diseases (CVD) and is mainly caused by atherosclerosis (1). It is the primary cause of mortality in the majority of countries across the globe (17.8 million deaths annually) (2) and Iran (46% of all deaths) (3). By 2030, CAD is expected to become the most critical and prevalent issue that poses a threat to human health (4). Atherosclerosis builds up inflammatory cells, lipids accumulation, oxidative stress (OS), and extracellular matrix (ECM) in the inner lining of artery walls and encourages the growth of intimal smooth muscle cells (SMC). This can make arteries stiff and restrict blood flow, which can eventually cause heart failure, myocardial infarction (MI), and death in those affected (5, 6). Among the CADs, people who have coronary artery block above 50% (CAB > 50%) have a higher risk of mortality (7). Traditional cardiovascular risk factors do have a role in the pathogenesis of CAD, but other unique risk factors may be also at play (2). Various risk factors, including chronic inflammation, age, gender, genetics, obesity, diabetes mellitus (DM), high blood pressure, elevated blood lipids and disturbances of these metabolisms, smoking, OS, a sedentary lifestyle, and trace elements, influence it (8–11). While the main result is not completely clear and needs further study.

Previous studies have shown that PCSK9 may decrease circulating levels of IL-6 and other cytokines. However, it has also been reported that IL-6 inhibition can decrease circulating PCSK9 protein levels, potentially indicating a bidirectional relationship. Furthermore, the hypothesis that PCSK9 ameliorates low-grade inflammation at the local level within the vasculature without affecting systemic inflammation has been included. Additionally, PCSK9 is able to counteract the deleterious effects of IL-6 on the activation of inflammatory pathways, the instigation of autophagy, and the induction of oxidative stress. A positive correlation between PCSK9 and IL-1β, IL-6, and LC3B2/1 has been demonstrated, suggesting that higher PCSK9 expression correlates with elevated levels of these inflammatory and autophagy markers (12).

OS happens when the production of reactive nitrogen species (RNS) and reactive oxygen species (ROS) is out of balance and the body’s antioxidant defense system isn’t strong enough (13). Elevated levels of inflammatory cells, cytokines, and high-sensitivity C-reactive protein (hs-CRP) have been associated with inflammation, a key pathophysiological mechanism in the development of atherosclerosis (14). Chronic inflammation with OS influences vascular homeostasis, which includes endothelial and SMC growth, proliferation, and apoptosis (15). Superoxide dismutases (SOD) are metalloenzymes that metabolize superoxide to hydrogen peroxide and oxygen; they operate as the primary step in protection against ROS (16). Catalase (CAT) and glutathione peroxidase (GPx) break down hydrogen peroxide into water and oxygen. Myeloperoxidase (MPO) creates ROS intermediates, such as hypochlorous acid (HOCl), which is released by neutrophils and monocytes at sites of inflammation (11). Excessive ROS causes lipid damage and produces oxidized low-density lipoprotein (ox-LDL) in the sub-intimal region. Foam cells arise when scavenger receptors internalize ox-LDL within macrophages in the arteries. Ox-LDL also makes SMCs multiply and has chemotactic and cytotoxic effects on endothelial and monocyte cells, which speeds up the development of atherosclerosis (11, 17). Free radicals attack the polyunsaturated fatty acid during lipid peroxidation, forming malondialdehyde (MDA) and 4-Hydroxynonenal as a marker of the OS effect in lipids (18). The total antioxidant capacity (TAC) of plasma and body fluids signifies the cumulative activity of all antioxidant groups (19).

Hepatocytes synthesize the proprotein convertase subtilisin/kexin type 9 (PCSK9), which regulates cholesterol levels (20). PCSK9 triggers an autocrine action by binding to the low-density lipoprotein receptor (LDLR), causing the complex to undergo endocytosis and degradation in lysosomes within hepatocytes. This process leads to a decrease in LDLR at the cell membrane and an increase in low-density lipoprotein cholesterol (LDL-C) in blood (21, 22). Recent research has shown that PCSK9 not only regulates LDL-C concentration but also facilitates platelet activation and coagulation, and increase Inflammation, OS, and endothelial dysfunction (20, 23), that increase risk of MI incidence. In a prospective investigation of people with CAD, researchers found a positive relationship between PCSK9 concentration and the development and severity of CAD (24). In 2023, Sotlar and collaborators showed that the main way that PCSK9 affects CAD is through a rising concentration of atherogenic lipids (22). Furthermore, research employing individuals with CAD revealed a direct relationship between PCSK9 levels and the onset and intensity of CAD (25).

However, research from recent years has demonstrated that half of patients whose LDL-c and high-density lipoprotein-cholesterol (HDL-C) levels are normal have hazardous plaque buildup in their arteries (1, 26), therefore alternative risk of MI incidence is evaluated. Researchers looked into the link between PCSK9 and CVD by looking at how PCSK9 affects the metabolism lipids and levels of cholesterol and LDL-C (27), but inflammation and OS were almost ignored. PCSK9 is a practical treatment for the prevention and control of CVD. Indeed, high-sensitivity cardiac troponin I (hs-cTnI) serves as a biomarker and the gold standard for detecting myocardial injury and MI in patients, but this has less sensitivity for detecting cardiomyopathies (28). Based on studies, patients with the highest levels of PCSK9 in their plasma were 2.62 times more likely to experience recurrent vascular events compared to those with lower levels of PCSK9 in their plasma (29). These findings were supported by experiments where recombinant human PCSK9, when added to healthy human platelet-rich plasma, was able to significantly increase platelet aggregation and reduce the aggregation lag time after stimulation with a subthreshold concentration of epinephrine (0.3 and 0.6 mM) (30). Similarly, platelets incubated with PCSK9, at the concentration found in the circulation of 1 patients with atrial fibrillation (AF), increased platelet aggregation and the release of thromboxane B2 (TxB2) (31), which is a marker of *in vivo* platelet activation (23, 32).

These are the first studies of their kind to look at how PCSK9 is related to heart parameters like hs-cTnI, EF%, ox-LDL, and hs-CRP, as well as OS parameters like TAC, SOD, CAT, GPx, MPO, and MDA in people who have CAB > 50%. Furthermore, we evaluated cardiac-related parameters for the best sensitivity and specificity for the diagnosis of CAB > 50%.

## Materials and methods

### Subjects and data collection

This case-control study was carried out at Tehran Heart Centre Hospital (October-August 2023, n = 126). A cardiologist confirmed that 63 of them had confirmed CAB > 50% through coronary angiography. An additional 63 control subjects had normal coronary arteries. The power analysis was conducted with the following parameters: Significance Level (α): 0.05, Statistical Power (1-β): 0.8, Effect Size: 0.5, Standard Deviation (σ): 1, Expected Difference in Means (Δ): 0.5 (n=((1.96 + 0.84)ˆ2·2)/0.5^2), n=((2.8)2·2/0.25), n=(7.84·2/0.25), n=(15.68/0.25), n=62.72).

Thus, the required sample size was determined to be 63 participants per group. We excluded individuals with underlying cardiovascular diseases (CVDs), cerebrovascular diseases, carotid artery stenosis, a history of myocardial infarction (MI), cancer, liver and kidney diseases, and autoimmune and inflammatory diseases. Participants who had taken antilipemic drugs or dietary supplements (e.g., vitamin E, vitamin D, and vitamin C) within one month before the study were also excluded. To ensure compliance with the exclusion criteria, detailed medical histories were obtained from all participants through structured interviews conducted by trained physicians. Additionally, medical records were reviewed to verify past diagnoses, medication use, and supplement intake. In cases of uncertainty, participants were asked to provide further clarification regarding their health status and medication history.

### Ethics and consent

The researchers collected participant data through a questionnaire, while strictly adhering to the ethical guidelines outlined in the *Declaration of Helsinki*. The Tehran University of Medical Sciences Ethics Committee (IR.TUMS.MEDICINE.REC.1402.047) granted ethical approval, and all participants provided their informed consent.

### Biochemical and demographic parameters

Various biochemical and anthropometric parameters have been collected from the participants in this study. The researchers calculated the body mass index (BMI) and EF% by employing the conventional formula: BMI = weight (kg)/height (m2) and EF% = stroke volume/end-diastolic volume *100. The physician used a standard sphygmomanometer to record participants’ systolic and diastolic blood pressure (SBP and DBP) measurements while they were seated. Following aseptic precautions 5 ml of fasting blood sample was drawn in red top tubes that were later centrifuged at 4000 rpm for 7 minutes to obtain the supernatant serum. The serum was separated and aliquoted and stored at -80 degree C until analysis. (Mindray BS200, China) and commercially available kits (Delta Darman Part, Iran) were used to assess biochemical parameters, including LDL-C, HDL-C, total cholesterol (TC), triglycerides (TG), very low-density lipoprotein (VLDL), fasting blood glucose (FBG), HbA1c (glycated hemoglobin), creatinine, urea, and albumin, following the manufacturer’s guidelines.

### Oxidative stress parameters

OS parameters, such as TAC, MDA, MPO activity, SOD activity, CAT activity, and GPx activity, were measured using colorimetric method kits (NAVAND salamat/Iran).

### ELISA and chemiluminescent assays

The enzyme-linked immunosorbent assay (ELISA) kit used to conduct the assay of PCSK9 and ox-LDL was obtained from Wuhan Feiyue, China (E23ONI609):. hs-cTnI and hs-CRP were measured using a chemiluminescent immunometric assay kit from Aldeal Lab-Tech, Cyprus (E23OAY398).

### Statistical analysis

The qualitative parameters have been represented as numbers (%), whereas the quantitative parameters have been provided as means ± standard errors of the mean (mean ± SEM). We determined data normality using the Kolmogorov-Smirnov test. Group differences were determined using specific analyses, such as Chi-square or exact Fisher’s tests for qualitative parameters and one-sample t-tests or Mann-Whitney U tests for the quantitative parameters. Data correlations were explored using Spearman’s test. hs-CTnI frequency levels have been changed from asymmetrical distributions to ln to improve the linear model’s normality assumption. The logistic regression analysis examined the relationship between CAB > 50% and various parameters. The analysis included both crude and adjusted models, with adjustments made for age, BMI, and gender. Risk ratios (RR) of disease (CAB > 50%) are calculated by dividing the data into quartiles (Q1, Q2, Q3, and Q4), using the 1st quarter (Q1) as the reference for comparison. The Receiver Operating Characteristic (ROC) with an Area Under the Curve (AUC) at 95% Confidence Interval (CI) was used to figure out how useful the parameters were for diagnosing CAB > 50%. The Youden index was employed to calculate cut-off levels for each parameter based on their highest sensitivity and specificity. The analysis was conducted using SPSS software version 27, and graphs were drawn using GraphPad Prism software (version 9). Statistical significance was assessed using a significance value of *p* < 0.05.

## Results

### Demographic and biochemical parameters

**Table 1** provides details of the demographic and biochemical parameters. Age (P = 0.002), SBP (P = 0.01), BMI (P = 0.01), FBG (P = 0.008), HbA1c (P = 0.02), and TC (P = 0.02) were higher in the CAB > 50% group compared to controls. There were no notable differences observed in gender, DBP, TG, LDL-C, HDL-C, VLDL, creatinine, urea, and albumin amongst the groups being studied.

**Table 1:** Demographic and biochemical parameters of CAB **>**50% and control.

Variables		Control (n=63)	CAD > 50% (n=63)	P-value
Age (years) Mean ± SEM		51.65 ± 1.23	58.38 ± 1.26	0.002
Gender number, (%)	Female	21 (33.33)	12 (19.04)	0.06
	Male	42 (66.66)	51 (80.95)	
Blood pressure (mmHg) Mean ± SEM	DBP	74.25 ± 1.32	78.09 ± 1.62	0.06
	SBP	121.32 ± 1.22	126.34 ± 1.61	0.01
BMI (kg/m^2^) Mean ± SEM		24.75 ± 0.32	26.82 ± 0.72	0.01
FBG (mg/dl) Mean ± SEM		115.8 ± 2.44	125.6 ± 2.08	0.008
HbA1c (%) Mean ± SEM		5.74 ± 0.15	6.32 ± 0.22	0.02
TC (mg/dl) Mean ± SEM		154.22 ± 3.67	168.17 ± 3.06	0.02
TG (mg/dl) Mean ± SEM		153.9 ± 5.2	153.43 ± 8.27	0.87
HDL-C (mg/dl) Mean ± SEM		51.28 ± 1.94	55.96 ± 2.24	0.22
LDL-C (mg/dl) Mean ± SEM		78.38 ± 2.37	81.53 ± 2.73	0.5
VLDL (mg/dl) Mean ± SEM		30.78 ± 1.04	30.68 ± 1.65	0.97
Creatinine (mg/dl) Mean ± SEM		0.93 ± 0.03	1.08 ± 0.05	0.09
Urea (mg/dl) Mean ± SEM		39.06 ± 1.29	41.45 ± 2.43	0. 1
Albumin (mg/dl) Mean ± SEM		4.18 ± 0.1	4.21 ± 0.09	0.59

BMI, Body Mass Index; SBP, Systolic Blood Pressure; DBP, Diastolic Blood Pressure; FBG, fasting blood glucose; HbA1c, Glycated hemoglobin; TC, Total Cholesterol; TG, Triglycerides; HDL-C, High-Density Lipoprotein-cholesterol; LDL-C, Low-Density Lipoprotein-cholesterol; VLDL, very-low-density lipoprotein.

Mean ± SEM and number (%) are used to present the parameters. One-sample t-tests or Mann-Whitney U tests were used to analyze quantitative data, and a chi-square test was used to analyze qualitative data.

### Oxidative stress parameters

The healthy subjects exhibited a higher serum concentration of TAC (*p* < 0.001), GPx (*P* = 0.03), CAT (*p* < 0.001), and SOD (*p* < 0.001) activity compared to the CAB > 50% group. Alternatively, the findings indicated that those with a CAB > 50% exhibited elevated levels of MPO and MDA (*p* < 0.001).

### MPO, MDA, TAC, GPx, CAT, and SOD activity

**Figure 1:**
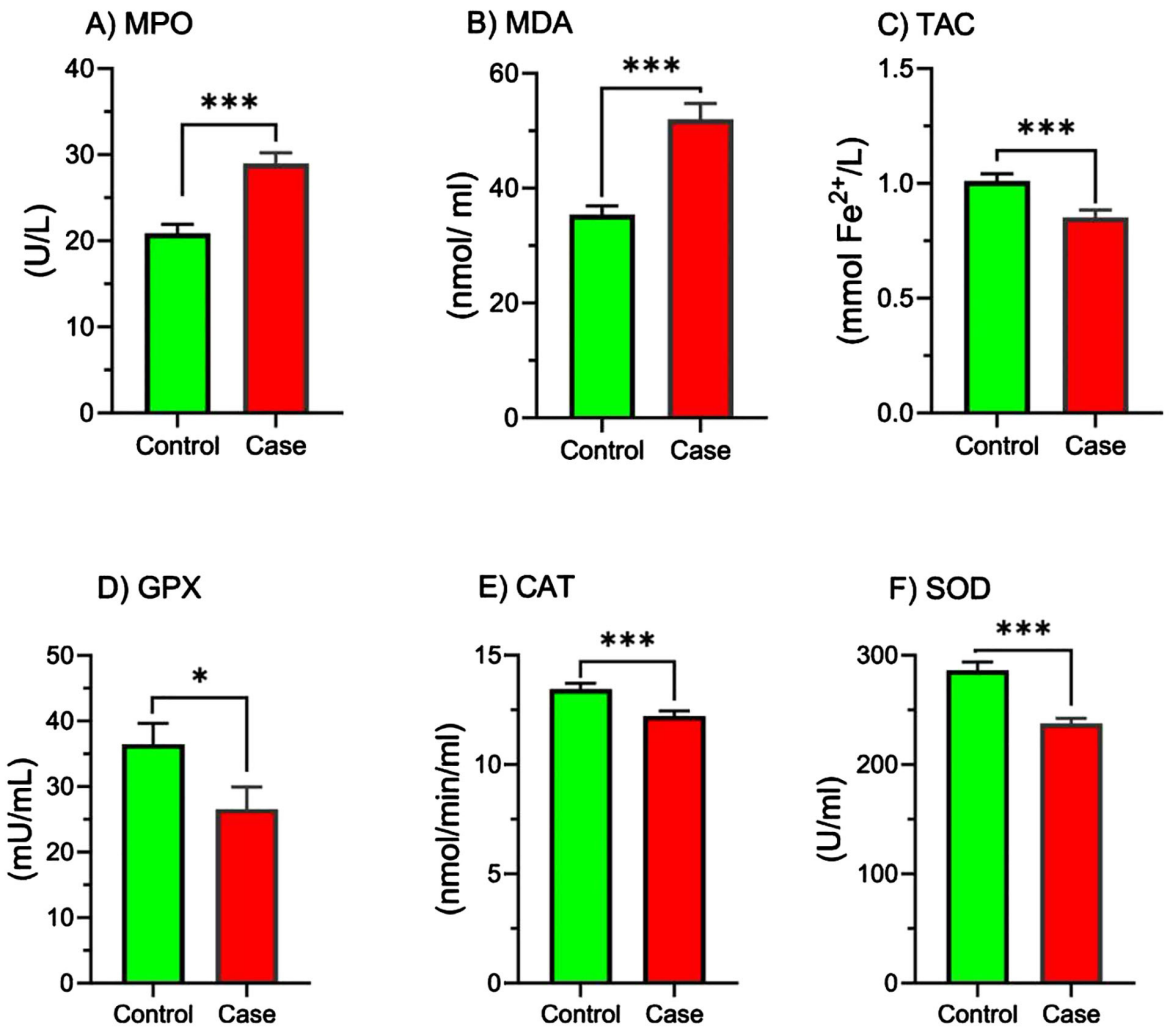
Comparison of MPO, MDA, TAC, GPx, CAT, and SOD activity in study groups. Comparison of oxidative stress markers and antioxidant enzyme activities between the control and case groups. Bar graphs illustrate the levels of **(A)** myeloperoxidase (MPO), **(B)** malondialdehyde (MDA), **(C)** total antioxidant capacity (TAC), **(D)** glutathione peroxidase (GPX), **(E)** catalase (CAT), and **(F)** superoxide dismutase (SOD) in the control (green bars) and case (red bars) groups. MPO, Myeloperoxidase; MDA, Malondialdehyde; TAC, Total antioxidant capacity; GPx, Glutathione Peroxidase; CAT, Catalase; SOD, Superoxide dismutase. Parameters are presented as mean ± SEM. One-sample t-tests or Mann-Whitney U tests were used to analyze data, and the significance level is as: *p < 0.05, ***p < 0.001.

**Figure 1** illustrates the comparison of MPO, MDA, and TAC levels, as well as the activity of GPx, CAT, and SOD enzymes, between the control and study groups. The results indicate that the study group exhibited significantly higher levels of MPO (**Figure 1A**, p < 0.001) and MDA (**Figure 1B**, p < 0.001) compared to the control group. Conversely, the total antioxidant capacity (TAC) was significantly lower in the study group (**Figure 1C**, p < 0.001).

Furthermore, the activity of the antioxidant enzymes GPx (**Figure 1D**, p < 0.05), CAT (**Figure 1E**, p < 0.001), and SOD (**Figure 1F**, p < 0.001) was significantly reduced in the study group.

### EF%, hs-CRP, ox-LDL, hs-CTnI, and PCSK9 levels

**Figure 2:**
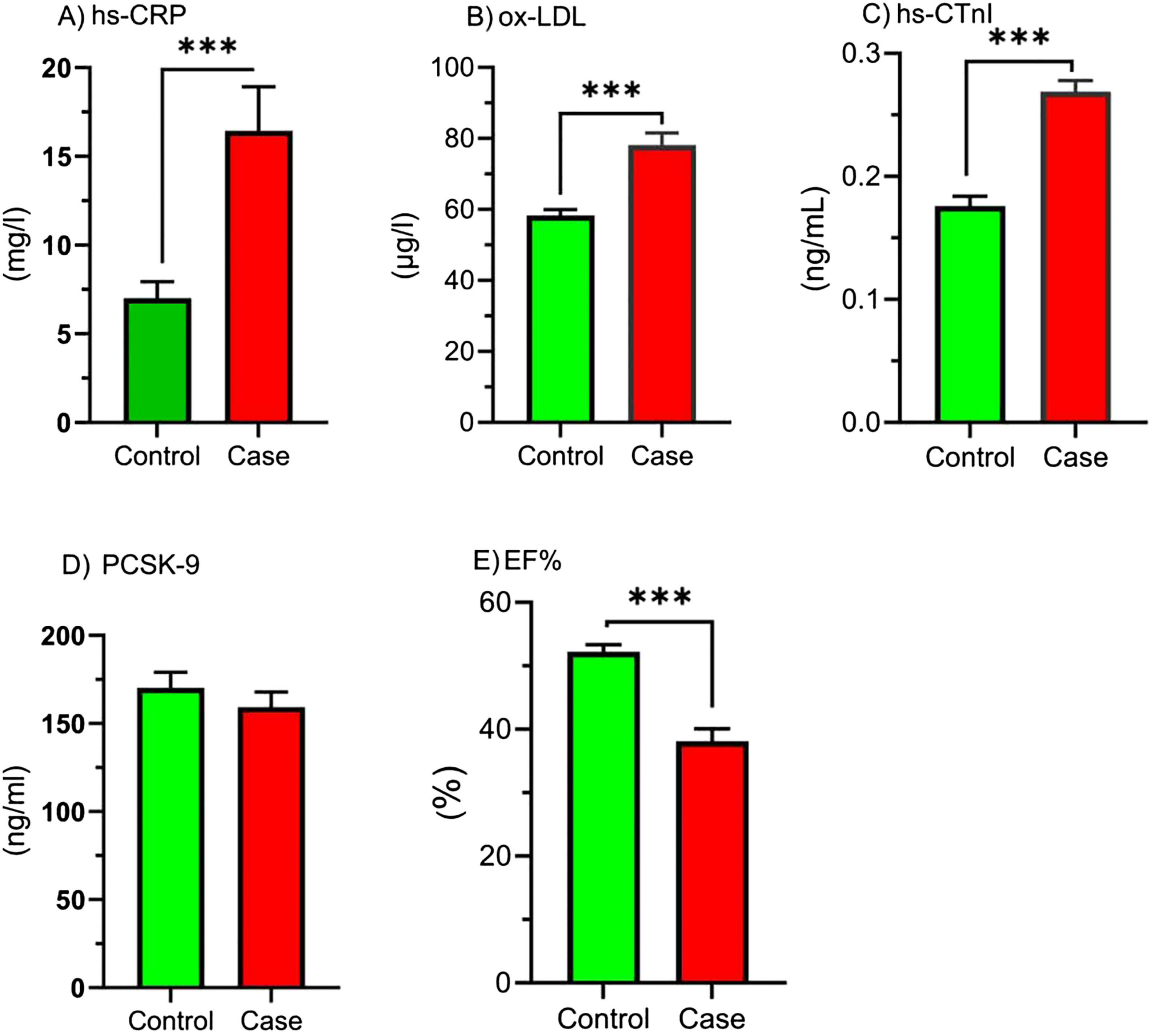
Comparison of hs-CRP, ox-LDL, hs-CTnI, PCSK9, and EF% in study groups. Comparison of inflammatory markers, oxidized LDL, cardiac marker, PCSK-9, and ejection fraction between control and case groups. Bar graphs show the levels of **(A)** high-sensitivity C-reactive protein (hs-CRP), **(B)** oxidized low-density lipoprotein (ox-LDL), **(C)** high-sensitivity cardiac troponin I (hs-cTnI), **(D)** proprotein convertase subtilisin/kexin type 9 (PCSK-9), and **(E)** ejection fraction (EF%) in the control (green bars) and case (red bars) groups. Parameters are presented as the mean ± SEM. One-sample t-tests or Mann-Whitney U tests were used to analyze the data, and the significance level is as follows: *** *p* < 0.001. hs-CRP, High-sensitivity C-reactive Protein; ox-LDL, Oxidized low-density lipoprotein; hs-CTnI, High-sensitivity cardiac troponin I; PCSK9, proprotein convertase subtilisin/kexin type 9; EF %, Ejection fraction percentage.

**Figure 2** elucidates the concentrations of hs-CRP, ox-LDL, hs-CTnI, PCSK9, and EF% levels in study groups. Based on these, hs-CRP, ox-LDL, and hs-CTnI have been greater in the CAB > 50% relative to the control (*p* < 0.001). The EF% was lower in the CAB > 50% group compared to the control (*p* < 0.001). Furthermore, no difference was shown in PCSK9 levels.

### Correlation coefficient

**Table 2** displays the correlation coefficients ranging from -0.39 to 0.55, obtained from Spearman’s correlation assessment aimed at ascertaining the link between variables. According to the table, EF% exhibits a positive link with SOD and a negative correlation with MDA, MPO, ox-LDL, hs-cTnI, and hs-CRP (*p* < 0.05). hs-cTnI has a positive correlation with MDA, MPO, and ox-LDL and a negative correlation with CAT, GPx, and SOD (*p* < 0.05). ox-LDL exhibits a positive correlation with MPO and an inverse correlation with TAC, CAT, and GPx (*p* < 0.05). MDA has a negative association with TAC and SOD (*p* < 0.05). SOD is directly associated with TAC and CAT (*p* < 0.05). hs-CRP has a negative correlation with TAC, and GPx has a positive correlation with TAC (*p* < 0.05). Indeed, MPO has a negative relationship with CAT, and PCSK9 positively related to MDA (*p* < 0.05).

**Table 2:** Spearman**’**s Correlation.

Parameters	TAC	CAT	GPx	SOD	MDA	MPO	ox-LDL	hs-cTnI	PCSK9	hs-CRP	EF %
TAC	1	0.09	0.19^*^	0.16^*^	-0.27^**^	-0.11	-0.20^*^	-0.16	-0.13	-0.25^**^	0.08
CAT		1	0.13	0.21^*^	-0.06	-0.24^**^	-0.24^*^	-0.26^**^	0.04	-0.1	0.05
GPx			1	0.06	-0.11	-0.09	-0.22^*^	-0.19^*^	0.07	-0.86	0.11
SOD				1	-0.18^*^	-0.1	-0.19	-0.39^**^	-0.04	-0.14	0.26^**^
MDA					1	0.14	0.1	0.19^*^	0.24^*^	0.09	-0.24^*^
MPO						1	0.55^**^	0.19^*^	0.19	0.07	-0.17^*^
ox-LDL							1	0.25^*^	0.13	0.08	-0.29^**^
hs-cTnI								1	-0.01	0.12	-0.34^**^
PCSK9									1	0.06	0.04
hs-CRP										1	-.22^*^
EF %											1

TAC, Total antioxidant capacity; CAT, Catalase; GPx, Glutathione Peroxidase; SOD, Superoxide dismutase; MDA, Malondialdehyde; MPO, Myeloperoxidase; ox-LDL, Oxidized low-density lipoprotein; PCSK9, Proprotein convertase subtilisin/kexin type 9; hs-CRP, High-sensitivity C-reactive Protein; EF %, Ejection fraction percentage.

Spearman’s rho test was performed to examine the correlation between variables, and the significance is as follows:

The correlation is significant at the 0.01 level (2-tailed) **

The correlation is significant at the 0.05 level (2-tailed) *

### Logistic regression

We conducted a logistic regression analysis based on quartiles, with Q1 as the reference for the other quartiles, to determine the impact of increasing or decreasing parameters on the risk of disease. This approach is particularly useful when parameters, such as oxidative stress markers (MPO and ox-LDL), lack established reference values or clinical thresholds. A logistic regression analysis was conducted to assess the severity of the disease (CAB > 50%). Data categorized into quartiles, specifically Q1, Q2, Q3, and Q4. The first quarter served as a reference for determining and establishing the risk ratio (RR) of the CAB > 50%. **Table 3** displays the result. Adjusted TAC in 4th quartiles (TAC-Q4, RR = 0.13, *P* = 0.02), adjusted SOD in 4th quartiles (SOD-Q4, RR = 0.09, *P* = 0.003), adjusted CAT in 3rd and 4th quartiles (CAT-Q3, RR = 0.09, *P* = 0.005; CAT-Q4, RR = 0.05, *P* = 0.003), and adjusted GPx in the 2nd, 3rd, and 4th quartiles (GPx-Q2, RR = 0.06, *P* =0.003; GPx-Q3, RR = 0.08, *P* = 0.006; GPx-Q3, RR = 0.2, *P* = 0.04) reduce risk of disease. While adjusted EF% in all of the quartiles (EF%-Q2, RR = 0.07, *p* < 0.001; EF%-Q3, RR = 0.03, *p* < 0.001; EF%-Q4, RR = 0.01, *p* < 0.001) decrease the risk of diseases. Adjusted ln-hs-cTnI in 3rd and 4th quartiles (hs-cTnI-Q3, RR = 2.86, P = 0.04, hs-cTnI-Q4, RR = 6.29, *p* < 0.001), and adjusted ox-LDL in 3rd and 4th quartiles (ox-LDL-Q3, RR = 32.93, P = 0.008, ox-LDL-Q4, RR = 67.36, P = 0.004) elevate the risk of diseases. Furthermore, adjusted MDA in all quartiles (MDA-Q2, RR = 14.18, P = 0.03; MDA-Q3, RR = 30.31, P = 0.004; MDA-Q4, RR = 61.29, P = 0.001), adjusted MPO in all quartiles (MPO-Q2, RR = 7.38, P = 0.01; MPO-Q3, RR = 15.42, *p* < 0.001; MPO-Q4, RR= 0.01; hs-CRP-Q4, RR = 14.5, *p* < 0.001) increased risk of diseases. Also, adjusted PCSK9 in 2nd quartiles (PCSK9-Q2, RR = 7.89, *p* = 0.002) increases the risk of disease.

**Table 3:** Logistic regression test to evaluate the severity of the disease.

Parameters	Quartile	Crude RR	CI-95%	P-value	Adjusted RR	CI-95%	P-value
TAC (mmol Fe2+/L)	Q1 < 0.78	0.06	0.1-0.34	0.002	0.13	0.007-2.45	0.007
	Q2: 0.78 - 0.93	Ref			Ref		
	Q3: 0.93 - 1.05	0.47	0.16-1.36	0.16	0.91	0.2-4.02	0.9
	Q4 > 1.05	0.47	0.17-1.32	0.15	0.62	0.15-2.52	0.5
		0.22	0.07-0.67	0.008	0.13	0.02-0.82	0.02
SOD activity (U/ml)	Q1 < 229.3	0.097	0.96-0.98	<0.001	0.97	0.96-0.99	0.005
	Q2: 229.3 - 251.59	Ref			Ref		
	Q3: 251.59 - 297.16	3.82	1.15-12.71	0.02	3.91	0.99-15.48	0.5
	Q4 > 297.16	0.76	0.27-2.11	0.6	0.72	0.22-2.35	0.58
		0.08	0.02-0.34	<0.001	0.09	0.01-0.45	0.003
CAT activity (nmol/min/ml)	Q1 < 11.32	0.71	0.58-0.87	0.001	0.57	0.38-0.87	0.01
	Q2: 11.32 - 12.7	Ref			Ref		
	Q3: 12.7 - 14.2	0.31	0.1-0.93	0.03	0.24	0.05-1.09	0.06
	Q4 > 14.2	0.33	0.1-0.93	0.03	0.09	0.19-0.49	0.005
		0.18	0.06-0.55	0.003	0.05	0.1-0.33	0.003
GPx activity (mU/ml)	Q1 < 16.48	0.098	0.96-0.99	0.04	0.99	0.98-1.02	0.08
	Q2: 16.48 - 24.03	Ref			Ref		
	Q3: 24.03 - 35.18	0.09	0.02-0.36	<0.001	0.06	0.09-0.39	0.003
	Q4 > 35.18	0.04	0.01-0.18	<0.001	0.08	0.01-0.5	0.006
		0.1	0.02-0.36	<0.001	0.2	0.04-0.98	0.04
MPO (U/L)	Q1 < 17.77	1.12	1.06-1.19	<0.001	1.21	1.1-1.34	<0.001
	Q2: 17.77 - 23.7	Ref			Ref		
	Q3: 23.7 - 30.31	4.8	1.42-16.18	0.01	7.38	1.49-36.5	0.01
	Q4 > 30.31	9.16	2.73-30.68	<0.001	15.42	3.06-77.71	<0.001
		16	2.24-60.31	<0.001	59.31	8.96-392.2	<0.001
MDA (nmol/ml)	Q1 < 32.07	1.11	1.05-1.14	<0.001	1.1	1.02-1.18	0.007
	Q2: 32.07 - 40.89	Ref			Ref		
	Q3: 40.89 - 51.42	3.89	1.1-13.68	0.03	14.18	1.28-156.41	0.03
	Q4 > 51.42	8.05	4.42-26.7	<0.001	30.31	2.94-312.2	0.004
		26.45	6.29-111.2	<0.001	61.19	4.8-733.2	0.001
ln-hs-CTnI (ng/ml)	Q1 < 0.156	2.99	10.03-18.21	<0.001	4.33	0.84-0.95	<0.001
	Q2: 0.156 - 0.208	Ref			Ref		
	Q3: 0.208 - 0.301	1.4	0.73-7.89	0.14	1.7	0.76-42.5	0.09
	Q4 > 0.301	2.13	2.43-28.35	<0.001	2.86	1.06-74	0.04
		4.83	5.94-121.08	<0.001	6.29	7.11-618	<0.001
ox-LDL (µg/l)	Q1 < 57.78	1.15	1.07-1.22	<0.001	1.23	0.81-0.94	<0.001
	Q2: 57.78 - 64.87	Ref			Ref		
	Q3: 64.87 - 72.97	3.75	0.86-16.91	0.07	10.46	0.89-122	0.06
	Q4 > 72.97	10.66	2.5-45				

RR, risk ratio; 95% CI, Confidence interval; Abbreviation, TAC, Total antioxidant capacity; CAT, Catalase; GPx, Glutathione Peroxidase; SOD, Superoxide dismutase; MDA, Malondialdehyde; hs-CTnI, High-sensitivity cardiac troponin I; MPO, Myeloperoxidase; ox-LDL, Oxidized low-density lipoprotein; PCSK9, Proprotein convertase subtilisin/kexin type 9; hs-CRP, High-sensitivity C-reactive Protein; EF %, Ejection fraction percentage. A logistic regression test was performed to evaluate the severity of the disease (CAB > 50%). The data were presented crudely and adjusted for age, gender, and BMI. In addition, we expressed the data in quarters (Q1, Q2, Q3, and Q4) and compared the first quarter (Q1 ≤ 25%) to other quarters as a reference.

### ROC curve

**Figure 3:**
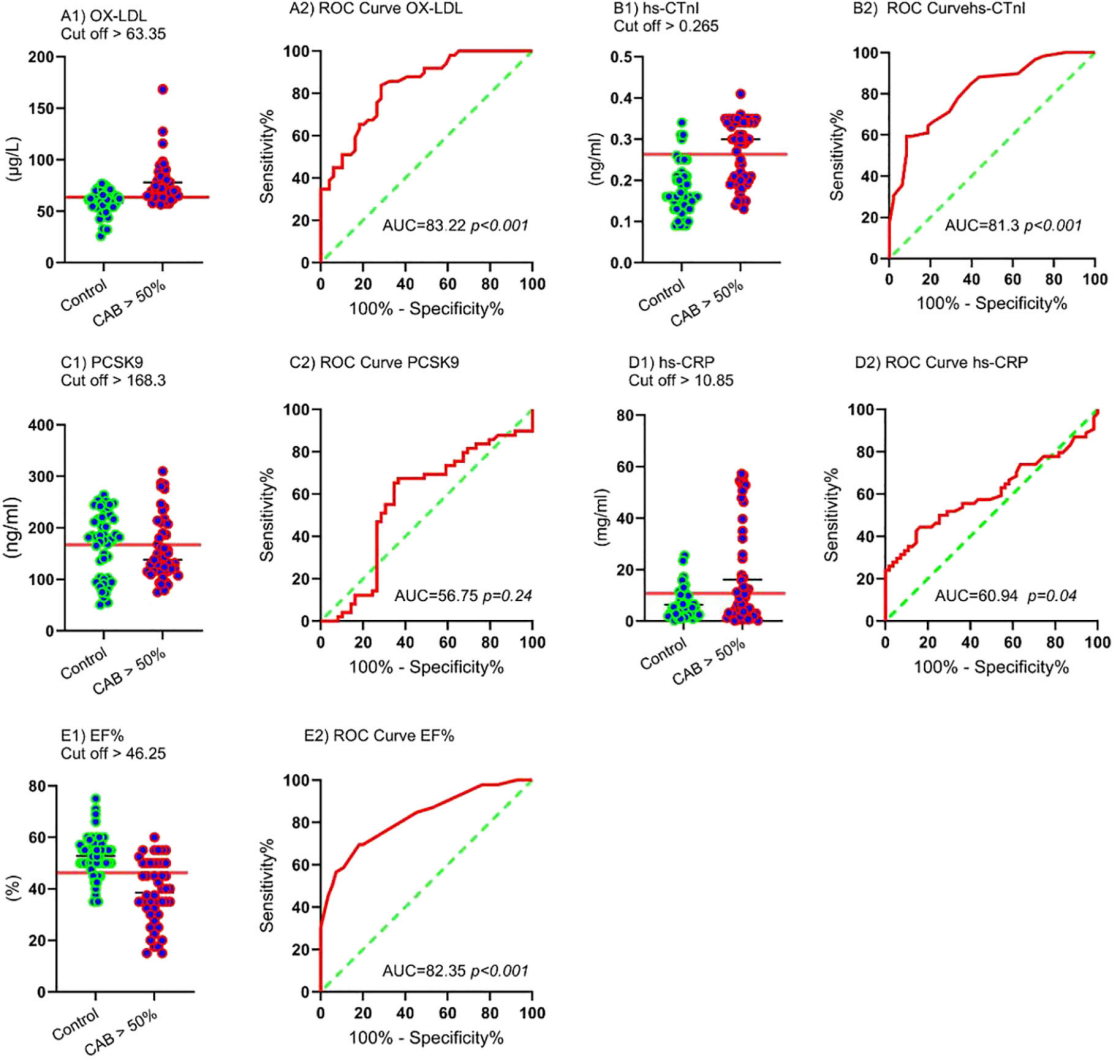
ROC curves and cut-off values for CAB > 50% ox-LDL: Cut-off **(A1)** and ROC curve **(A2)**, hs-CTnI: Cut-off **(B1)** and ROC curve **(B2)**, PCSK9: Cut-off **(C1)** and ROC curve **(C2)**, hs-CRP: Cut-off **(D1)** and ROC curve **(D2)**, EF%: Cut-off **(E1)** and ROC curve **(E2)**.

The AUC and ROC curve indicate the overall effectiveness of a test; the AUC for an optimal ROC curve is 100. ROC analysis determined the optimal cut-off with the highest specificity and sensitivity for MI (CAB > 50%) diagnostic parameters. **Figure 3(A1, A2, ETC)** and **Table 4** illustrate the results of the study. **Figure 3** displays the Receiver Operating Characteristic (ROC) curves and corresponding cut-off values for several diagnostic markers in relation to significant coronary artery blockage (CAB > 50%). The figure is organized into panels, each dedicated to a specific marker: ox-LDL (A1, A2), hs-CTnI (B1, B2), PCSK9 (C1, C2), hs-CRP (D1, D2), and EF% (E1, E2). For each marker, the left panel (A1, B1, C1, D1, E1) presents a scatter plot showing the distribution of marker levels in both control patients and those with CAB > 50%, with the optimal cut-off value indicated. The right panel (A2, B2, C2, D2, E2) displays the ROC curve itself, plotting sensitivity against 100% - specificity. The area under the curve (AUC) and its associated p-value are provided for each ROC curve, indicating the marker’s ability to discriminate between the two groups. The study determined the best AUC and the optimal cut-off value for variables in identifying the CAB > 50% categories as follows: ox-LDL (AUC = 83.22, cut-off > 63.35 μg/L, *p* < 0.001), EF% (AUC = 82.35, cut-off < 46.25%, *p* < 0.001), hs-cTnI (AUC = 81.3, cut-off > 0.265 ng/ml, *p* < 0.001), hs-CRP (AUC = 60.94, cut-off > 10.85 mg/ml, *p* = 0.04), and PCSK9 (AUC = 56.75, cut-off > 168.3 ng/ml, *p* = 0.24).

**Table 4:** AUC and cut-off for parameters related to the diagnosis of CAB > 50%.

Parameters	Case	AUC	Sensitivity	Specificity	Cut-Off	95% CI	P-value
ox-LDL	CAB > 50%	83.22	81.63	71.43	> 63.35 µg/L	0.75 to 0.90	<0.001
hs-CTnI	CAB > 50%	81.3	59.32	91.67	> 0.265 ng/ml	0.73-0.89	<0.001
PCSK9	CAB > 50%	56.75	63.27	67.35	> 168.3 ng/ml	0.44-0.68	0.24
hs-CRP	CAB > 50%	60.94	40.74	85.45	> 10.85 mg/ml	0.50-0.75	0.04
EF%	CAB > 50%	82.35	69.57	81.82	< 46.25%	0.74-0.90	<0.001

AUC assessment by ROC curve analysis and the best cut-off for parameters related to the diagnostic of CAB > %50 calculated with the highest sensitivity and specificity.

## Discussion

This study found that elevated MDA, MPO, hs-cTnI, hs-CRP, and ox-LDL increased the risk of CAB > 50% development, while higher TAC, SOD, GPx, CAT, and EF% **lowered** the risk of CAB > 50% development. However, there is no difference in PCSK9, LDL-C, and HDL-C levels, and they are associated with CAB > 50% development. EF% has a positive association with SOD and a negative association with MDA, MPO, ox-LDL, hs-cTnI, and hs-CRP. hs-cTnI has a direct link with MDA, MPO, and ox-LDL and an indirect link with CAT, GPx, and SOD. Indeed, ox-LDL has a direct correlation with MPO and an indirect correlation with TAC, CAT, and GPx. hs-CRP negatively associates with TAC, and PCSK9 has a positive correlation with OS. Furthermore, ox-LDL, EF%, and hs-cTnI have the highest AUC and sensitivity-specificity for detecting CAB > 50%.

PCSK9, alternatively referred to as neural apoptosis-regulated convertase-1 (NARC-1), was classified as the 9th member of the proteinase K subfamily when it was initially identified in 2003 (20, 33). The main function of PCSK9 in cholesterol regulation is to decrease LDLR on the surface of the hepatic membrane, increase LDL-C in circulation, and increase the risk of atherosclerosis and CVDs (22). PCSK9 has been implicated in activating platelets and causing thrombosis, altering lipid metabolism and increase LDL-C, increasing inflammation and activating inflammatory cells, promoting cell death through pyroptosis, autophagy, and apoptosis, raising levels of ROS and induce OS, and increase ox-LDL levels (20, 22, 23, 34). Based on these functions, PCSK9 inhibitors are used to reduce the risk of CVDs, atherosclerosis, and hypercholesterolemia in patients with recent MI and improve patient mortality. However power randomized controlled trial (RCT) studies are needed to prove this, and further surveys are needed (35). The results of these studies indicate that PCSK9 LDL-C, and HDL-C has no difference between the case and control, and there is positive correlation with MDA and OS. Almontashiri et al. reported elevated PCSK9 levels exclusively in acute myocardial infarction among CAD patients, while they remained unaltered in chronic myocardial infarction. Because CAD is a chronic condition, PCSK9 is not produced at elevated levels in all patients, but its production does increase during acute events. Additionally, some studies show that LDL-C and HDL-C levels are normal in patients and do not play a role in the incidence of atherosclerosis (1, 9, 36), indicating that other risk factors should be evaluated. Laugsand in the prospective population study showed PCSK9 levels increase the risk of MI in adjusted analysis with age and sex. Furthermore, they adjusted PCSK9 levels with LDL-C and other lipids observed to decrease the risk of MI (37). The findings of this study suggest that an increase in PCSK9 during acute conditions primarily influences the development of myocardial infarction (MI) and atherosclerosis through lipids and LDL-C. However, in chronic conditions where PCSK9 levels do not differ, other functions, such as oxidative stress (OS), are explored. According to Peng et al., PCSK9 levels in CAD plaques may act as a biomarker for detecting coronary artery disease (CAD) and can be linked to cardiac markers. However, the effectiveness of PCSK9 levels as a diagnostic marker for CAD remains uncertain (38). Also, ROC and AUC analyses indicated that PCSK9 is not worth investigating as a biomarker (AUC = 56.75, P = 0.24).

Inflammation and oxidative stress (OS) play a key role in the development of atherosclerosis and heart attacks (MI). This study found that MDA and oxidized LDL (ox-LDL) levels were higher in patients with coronary artery blockage (CAB) greater than 50%, while antioxidant systems like TAC, GPx, SOD, and CAT were less active. Research by Sagha et al. and Shiri et al. also shows increased OS in cases of CAB > 50%, along with higher nitric oxide and MDA levels, and lower TAC levels (1, 9). Furthermore, TAC (> 1.05 mmol Fe2+/L), SOD (> 297.16 U/ml), CAT (> 11.32 nmol/min/ml), and GPx (> 16.48 mU/mL) decrease the risk of disease, and MDA (> 32.07 nmol/ml) increases the risk of disease. During OS, ROS converts LDL-C to ox-LDL, is absorbed by macrophages, and then converted to foam cells (11). Mechanisms Involved in PCSK9’s Effects on OS: I) Impact on SIRT3: Research indicates that PCSK9i can counteract IL-6-induced inflammation, autophagy, and oxidative stress in endothelial cells. These protective effects are partially mediated by SIRT3; silencing SIRT3 reverses the benefits observed with PCSK9i treatment.

II) Effect on Autophagy: PCSK9i have been shown to reduce IL-6-induced autophagy in endothelial cells. This reduction is also mediated by SIRT3, as evidenced by the reversal of protective effects upon SIRT3 silencing. III) Effect on Oxidative Stress: PCSK9i can decrease mitochondrial ROS accumulation and improve mitochondrial membrane potential in endothelial cells. These effects are mediated by SIRT3, as silencing SIRT3 reverses the benefits of PCSK9i treatment. In summary, PCSK9i exhibit intrinsic anti-inflammatory, anti-autophagic, and antioxidant properties in endothelial cells, with these pleiotropic effects mediated, at least in part, by SIRT3. These findings provide new insights into the cardioprotective mechanisms of PCSK9i beyond LDL-cholesterol lowering (13). Also, The mechanism of PCSK9 in platelet activation is as follows: PCSK9 directly binds to the CD36 receptor on the platelet surface, enhancing platelet activation and downstream signaling, including Src and JNK kinases. Furthermore, PCSK9 increases ROS production through p38MAPK phosphorylation, leading to Nox2 activation, PLA2, arachidonic acid, and TxA2 signaling. Nox2-mediated ROS production increases ox-LDL formation, which amplifies platelet activation via both LOX1 and CD36 platelet receptors. All these events act as an amplifying signal for platelet activation, resulting in p-selectin expression, CD40L expression, and the release of granule contents (39).

PCSK9 enhances inflammation, oxidative stress (OS), and autophagy, though the exact mechanism is still unclear (12). Foam cells release inflammatory molecules and attractants like PDGF and TNF-α, which then activate vascular smooth muscle cells (VSMC), leading to the growth of the extracellular matrix (ECM) and the narrowing of arteries (6, 40). ROS-scavenging antioxidant systems correlate directly with EF% and with each other, and indirectly with ox-LDL, hs-CRP, and hs-cTnI. Increased myocardial infarction (MI) and myocardial damage lead to elevated hs-cTnI release. Studies by Muhammad et al. on aortic valve, mitral valve, and combined disease illustrated an increase in OS and related parameters, hs-cTnI and hs-CRP, an increase in the risk of disease, and a decrease in cardiac function (39). MPO and hs-CRP elevated in CAB > 50%, MPO (> 17.77 U/L), and hs-CRP (> 5.5 mg/l) increase the risk of disease. Indeed, they have positive correlations with hs-cTnI and negative correlations with TAC, SOD, and EF%. Neutrophils predominantly generate MPO, which is elevated in cases of MI and other inflammatory responses. Neutrophils secrete it into the extracellular fluid following OS and various inflammatory responses in MI (41). Mocatta et al. showed that MPO and OS are elevated in MI, and they increase the risk of MI and are negatively correlated with left ventricular ejection fraction (LVEF) (42). hs-CRP is a biomarker that is produced during the acute phase of inflammation. hs-CRP can influence the development of CADs via several mechanisms, including platelet and complement system activation, promotion of VSMC growth, macrophage activation, and lipid aggregation (43). In this study, hs-CRP boosted the risk of diseases and OS and reduced EF%. Also, ROC and AUC results reveal that hs-CRP (> 10.85 mg/ml) with an AUC of 60.94 is not good for CAB > %50 detection. But ox-LDL has the greatest AUC (AUC = 83.22, cutoff > 63.35 μg/L) for the diagnosis of CAB > %50.

This study indicated an EF% decrease in CAB > 50% and an EF% (> 37.5%) decrease in the risk of disease. An EF% > 50% is considered within the normal range. EF% < 40% suggests inadequate cardiac output and potential heart failure (44). Bauer et al. discovered a significant correlation between an EF% of 30 or lower with mortality in all patients throughout the subsequent five-year period (45). It was also shown that EF% was less than 47.25% with an AUC of 82.35 after ox-LDL, making it the best marker for detecting CAB > 50%. Furthermore, EF% has direct relationships with SOD and indirect relationships with MDA, MPO, ox-LDL, hs-cTnI, and MPO. hs-cTnI increases in CAB > 50% groups, and has positive associations with ox-LDL, MDA, and SOD and negative associations with CAT, GPx, and SOD. Cardiomyopathy and MI caused by OS are associated with signs such as chest pain, changes in the electrocardiogram, and a rise in cardiac biomarkers, including hs-cTnI (39). hs-cTnI serves as a biomarker and the gold standard for detecting MI in patients, but not specific for Cardiomyopathy (28). Based on this study, after ox-LDL and EF%, hs-cTnI (AUC = 81.3, cut-off > 0.265 ng/ml) was considered a biomarker for the detection of CAB > 50%. Indeed, FPG, HbA1c, BMI, and SBP elevated in CAB > 50% more than normal groups. Diabetic patients who do not have a prior history of CAD have an equal likelihood of developing CVDs. Nevertheless, diabetic people who have previously had an MI face a chance of more than 40% of encountering another MI (46). Psaty et al. found a direct association between SBP, DBP, pulse pressure, and the risk of developing MI and stroke. However, SBP demonstrated to be the most accurate indicator of cardiovascular events (47). Excess weight and elevated BMI can lead to lipid sequestration in the arteries. Arterial damage and obstruction, which impede the flow of blood to the heart, can result in a MI (48).

Furthermore, our study has both strengths and limitations. A key strength is its classification of myocardial infarction (MI) patients into those with significant coronary artery blockage (CAB > 50%) based on angiographic findings. We then measured PCSK9, other cardiac parameters, and oxidative stress markers in this CAB > 50% group, examining the interrelationships between these factors. Furthermore, we evaluated the diagnostic potential of ox-LDL, hs-CTnI, PCSK9, hs-CRP, and EF% as biomarkers for CAB > 50%. However, our study is limited by its relatively small sample size. We also lacked additional demographic and dietary information, as well as measurements of small dense LDL levels. Future research should investigate PCSK9 protein levels in both chronic and acute disease states, exploring their relationship with relevant signaling pathways and their influence on the frequency and severity of heart attacks and atherosclerosis. This will provide a more comprehensive understanding of PCSK9’s role in the pathophysiology of these conditions.

## Conclusion

Our research indicated that even though PCSK9 plays a role in the incidence of MI by increasing LDL-C, other functions such as oxidative stress (OS) and inflammation must also be considered. Therefore, to properly evaluate PCSK9 function, LDL-C, inflammation, and OS parameters should be taken into account. Notably, while hs-cTnI is the gold standard for MI diagnosis, our findings suggest that ox-LDL (AUC = 83.22, > 63.35 μg/L) and EF% (AUC = 82.35, < 46.25%) outperform hs-cTnI (AUC = 81.3, > 0.265 ng/ml) in diagnosing CAB > 50%. This highlights the potential clinical utility of ox-LDL and EF% in early diagnosis and risk stratification, suggesting that they could serve as valuable complementary biomarkers for identifying high-risk patients.

## Data Availability

The original contributions presented in the study are included in the article/supplementary material. Further inquiries can be directed to the corresponding authors.
